# Photoelastic analysis of stress generated by wires when conventional and self-ligating brackets are used: A pilot study

**DOI:** 10.1590/2176-9451.19.5.074-078.oar

**Published:** 2014

**Authors:** Guilherme Caiado Sobral, Mário Vedovello, Viviane Veroni Degan, Milton Santamaria

**Affiliations:** 1 MSc in Orthodontics, School of Dentistry - University of Araras (UNIARARAS); 2 Professor, Department of Orthodontics, UNIARARAS; 3 Professor, Postgraduate program in Orthodontics, UNIARARAS

**Keywords:** Orthodontic brackets, Dental arch, Corrective orthodontics

## Abstract

**OBJECTIVE:**

By means of a photoelastic model, this study analyzed the stress caused on conventional and self-ligating brackets with expanded arch wires.

**METHOD:**

Standard brackets were adhered to artificial teeth and a photoelastic model was prepared using the Interlandi 19/12 diagram as base. Successive activations were made with 0.014-in and 0.018-in rounded cross section Nickel-Titanium wires (NiTi) and 0.019 x 0.025-in rectangular stainless steel wires all of which made on 22/14 Interlandi diagram. The model was observed on a plane polariscope - in a dark field microscope configuration - and photographed at each exchange of wire. Then, they were replaced by self-ligating brackets and the process was repeated. Analysis was qualitative and observed stress location and pattern on both models analyzed.

**CONCLUSIONS:**

Results identified greater stress on the region of the apex of premolars in both analyzed models. Upon comparing the stress between models, a greater amount of stress was found in the model with conventional brackets in all of its wires. Therefore, the present pilot study revealed that alignment of wires in self-ligating brackets produced lower stress in periodontal tissues in expansive mechanics.

## Abstract (pt)

**OBJETIVO:**

o presente estudo analisou, por meio de um modelo fotoelástico, a distribuição das tensões geradas em braquetes convencionais e autoligáveis quando ativados com arcos expandidos.

**MÉTODOS:**

braquetes convencionais foram colados em dentes artificiais e, em seguida, foi confeccionado o modelo fotoelástico, utilizando como base o diagrama 19/12, de Interlandi. Foram feitas trocas sucessivas com fios de liga de níquel-titânio (NiTi) de secção circular 0,014" e 0,018" e de liga de aço de secção retangular 0,019" x 0,025", todos no diagrama 22/14 de Interlandi. A cada troca de fio, o modelo foi observado em polariscópio plano, na configuração de campo escuro, e fotografado. Foi feita a substituição por braquetes autoligáveis e repetido o experimento. A análise foi qualitativa, observando o local e o padrão da tensão das franjas nos dois modelos analisados.

**CONCLUSÕES:**

os resultados identificaram uma maior padrão de tensões das franjas na região do ápice de pré-molares em ambos os modelos analisados. Ao se comparar as tensões entre os modelos, observou-se uma maior quantidade de tensão nas franjas no modelo com braquetes convencionais em todos os fios utilizados no experimento. Portanto, o presente estudo mostrou que o alinhamento dos fios nos braquetes autoligáveis produz forças mais suaves nos tecidos periodontais nas mecânicas expansionistas.

## INTRODUCTION

Nowadays, orthodontists have many techniques and methods available for treatment planning. There is a great variety of brackets, with different prescriptions and forms that allow the orthodontist to individualize each case according to patient's needs.^1^

These needs make the scientific community endeavor to innovate in orthodontic appliances. Innovation, in turn, leads to better control of dental movement, given that one of the greatest challenges faced by the orthodontist is to come up with mechanical solutions to stimulate biological reactions of the periodontium without compromising treatment outcomes.^2^

Correct management of orthodontic forces depends on a series of factors, including friction generated between wires and brackets. In orthodontic sliding mechanics, friction poses clinical difficulties to the orthodontist. High levels of friction could decrease bracket efficiency, thereby reducing the speed of dental movement and hindering anchorage control.^3^

The concern of producing less friction, i.e., lower attrition between wires and brackets, contributed to the development of self-ligating brackets in which the tooth moves with the wires serving as a guide, since it does not involve the use of elastic ligatures which significantly increase friction between wires and the slot.^4^

The difference between conventional and self-ligating brackets system is the absence of elastic or metallic ligatures in the latter. In other words, brackets have a closing system that leaves the wire free inside the slot.^5^

One of the purposes of orthodontic mechanics is gaining space in the arch before alignment of crowded teeth. Including badly-positioned teeth in the wire without previous space gain leads to unwanted displacements of adjacent teeth.^6^ On the other hand, according to Damon,^5^ lower friction treatment provides transversal adaptation that prevents potential side-effects of alignment, thereby providing treatment of crowded teeth without previous mechanic space gain.

In addition to treating crowding cases, this transversal adaptation might be used in favor of the orthodontist. For instance, in cases aiming at transversal expansion of one or both arches, Maltagliati^6^ showed that treatment with self-ligating brackets significantly increased the transversal dimensions. This unique behavior of the self-ligating system in comparison to the conventional one seems to derive from lower friction associated with heat activated nickel-titanium wires of small diameter acting as adjuvant in treatment results.^6^

One of the methods used to study the way forces manifest on bodies is by means of photoelasticity. The principle of photoelasticity is based on the fact that most materials turn birefringent (separation of light into two rays with different velocity and refraction indexes) when subjected to mechanical stress.^7^^,^ 8

Birefringence is manifested by colored fringes in areas of induced stress. Orthodontic material reproduces resilience of the periodontium.^9^ Monochromatic tones are used for analysis of force quantity, while colored fringes provide more information on stress direction and distribution.^10^

By means of photoelasticity, the present study analyzed the stress caused on conventional and self-ligating brackets when combined with nickel-titanium wires.

## MATERIAL AND METHODS

### Photoelastic model

Only one photoelastic model was made. Initially, with conventional brackets (Kirium, Abzil Indústria e Comércio Ltda, São José do Rio Preto, Brazil) which were afterwards replaced by self-ligating brackets (Portia, Abzil Indústria e Comércio Ltda, São José do Rio Preto, Brazil) bonded with cyanoacrylate (Superbonder glue Loctite, Barueri, SP) to lower artificial teeth (B2-306, Kilgore-Nissin, Kilgore International, USA).

The photoelastic model of the lower arch was manufactured on a wax roller based on Interlandi 19/12 diagram.^11^ The wax was cut so as to have a constant thickness throughout the model. A mold was made with the wax pressed on a muffle furnace.

The wax was thus removed with hot water, detergent and Remox (Vipi, Pirassununga, Brazil). The epoxy flexible photoelastic resin (Polipox, Ind. e Com. Ltda, São Paulo, Brazil) was handled in accordance with the manufacturer's specifications and placed on the space created by the wax until teeth roots were completely submerged. After 72 hours, the model was removed from the mold.

### Plane polariscope characteristics

The polariscope was assembled with the following components: a light source, a polarizer, the Photoelastic model and the analyzer (Keyko). The camera (SX120 IS, Canon Inc., Tokyo, Japan) was mounted on a tripod and positioned in front of the analyzer. The photoelastic model was placed on a rotating platform with measurement markings to ensure consistency in placing the model.

**Figure 1:**
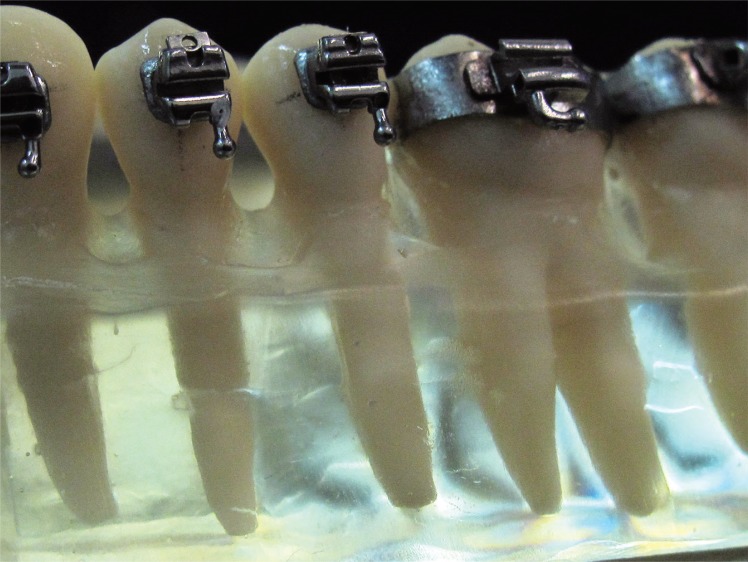
Photoelastic model without residual tension.

Prior to applying stress, the model was observed and photographed in frontal view, profile view (both left and right) and occlusal view. The objective was to assess absence of residual stress on the material and the initial conditions of the photoelastic resin (Fig 1).

### Mechanical trial

In conventional brackets with elastic ligatures, 0.014-in and 0.018-in rounded cross section Nickel-Titanium (NiTi) wires and 0.019 x 0.025-in rectangular stainless steel wires were successively placed in the 22/14 Interlandi diagram. The photoelastic model was made on the bases of dimensions corresponding to 19/12 Interlandi diagram. Thus, it aimed at exerting expansive forces during wire changes.

After each exchange of wire, the photoelastic model was photographed and analyzed for fringe standards. Self-ligating brackets were analyzed by the same means.

Photographs were taken based on the same criteria for both groups so as to avoid potential interference from other variables. All polariscope components remained within the same distance. Angling with the camera lenses and the photoelastic model also remained the same throughout the experiment.

In order to ensure that the model would be positioned in the exact same place after archwire placement, markings from the rotating platform were used. Photographs were taken at the same location under the same lighting conditions in the room.

### Qualitative assessment of photoelastic model 

In photograph analysis, the value of fringes depends on the type of material used, its width, length of light wave impacting and temperature of the model.^10^ Therefore, this study assessed - by qualitative means - stress distribution on photoelastic models.^12^

Qualitative assessment was carried out by assessing stress pattern on the model, expressed by different fringe colors on the root surface of premolars and marked in scores, as follows:

Results evolve from lack of stress (-) to a small whitish halo (+), a bigger white halo (++), followed by a yellow (+++), violet or magenta halo (++++) and a cyan or light blue halo(+++++). Assessment was conducted in the apical region and middle third of lower premolar roots.

Results are presented in tables according to groups, either with conventional or passive self-ligating brackets. Moreover, results were determined via descriptive statistics by categorizing the scores according to the colors of fringes.

## RESULTS

**Figure 2:**
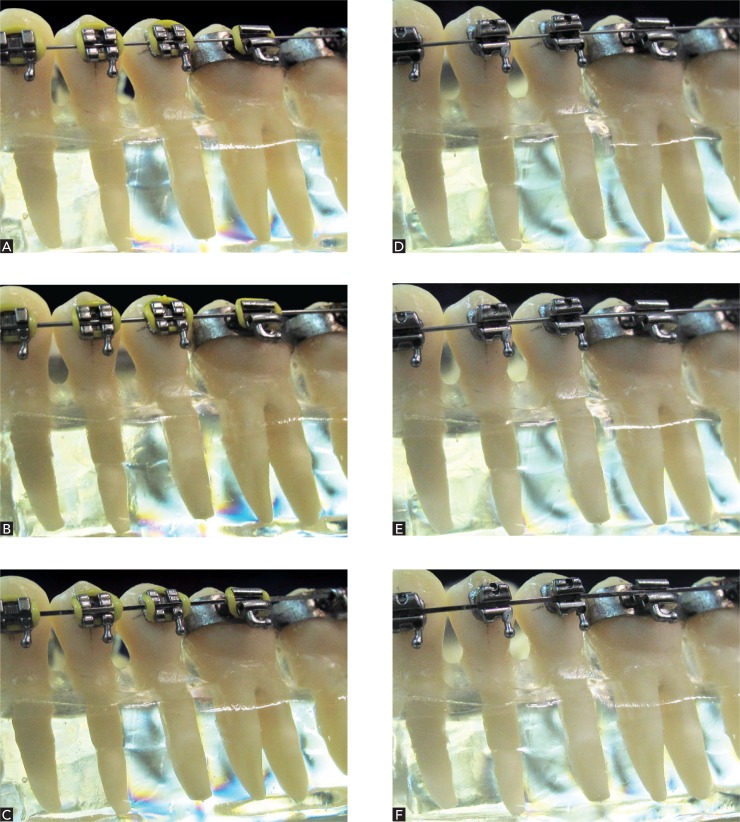
A, B, C) Conventional brackets; D, E, F) Self-ligating brackets.

Figure 2 illustrates photographs of premolars and molars under activation with conventional and self-ligating brackets. Figures A, B and C show the photoelastic model with conventional brackets associated to 0.014-in and 0.018-in NiTi wires as well as 0.019 x 0.025-in stainless steel wires respectively. Figures D, E and F show the photoelastic model with self-ligating brackets activated with 0.014-in and 0.018-in NiTi wires and 0.019 x 0.025-in stainless steel wires, respectively.

Results revealed that activations with either conventional or self-ligating brackets show the presence of stress in the apex of the premolars (Fig 2). However, conventional brackets produced higher stress due to greater concentration of blue and violet colored fringes, especially in activations with 0.018-in NiTi wires and 0.019 x 0.025-in stainless steel wires (Figs 2A, B and C; Table 1). In the middle third, stress was only found in activations with conventional brackets, as yellow fringes were found in the three activations (Figs 2A, B and C; Table 2). The same was not found in self-ligating brackets (Figs 2D, E and F; Table 2).

**Table 1:** Qualitative analysis of the apical region of premolar roots expressed in scores according to the colors of the fringes on the photoelastic model.

WIRE	SELF-LIGATING BRACKETS	CONVENTIONAL BRACKETS
0.014-in NiTi	++++	+++
0.018-in NiTi	+++++	+++
0.019 x 0.021-in Stainless steel	+++++	+++

**Table 2:** Qualitative analysis of the middle third of premolar roots expressed in scores according to the colors of the fringes on the photoelastic model.

WIRE	SELF-LIGATING BRACKETS	CONVENTIONAL BRACKETS
0.014-in NiTi	+++	-
0.018-in NiTi	+++	-
0.019 x 0.021-in Stainless steel	+++	-

## DISCUSSION

This study reveals that both self-ligating and conventional brackets produce photoelastic stress under conditions of alignment with expansive forces, since diagramming of wires was larger than the size of the photoelastic model dental arch. It also found that stress concentrated in the apical region of premolars in both models, but with greater stress concentration in conventional brackets models.

Furthermore, lower periodontal forces, seen in the photoelastic model, allow more physiological expansive treatment. Pandis^13^ conducted a study comparing the intercanine and intermolar distance after treatment with conventional and passive self-ligating brackets. In the self-ligating group, intermolar distance was greater. However, buccal tipping of lower incisors was the same in both groups.

The system of self-ligating brackets can increase buccal tipping of incisors and the transverse dimension of the maxilla and the mandible.^14^ However, in patients with muscular balance, buccal tipping of incisors might be desired and better controlled, thereby not changing patient's facial profile.

Table 1 compares the stress observed in the apical region of premolar roots subjected to conventional and self-ligating systems. Greater concentration of photoelastic stress is observed in conventional brackets.

Lower friction between the wires and brackets,^4^^,^ 6 associated with resilient heat-activated nickel-titanium wires^5^ produce lower periodontal stress, as seen in the photoelastic study mode, thereby favoring expansive mechanics in crowding resolution. This treatment modality is indicated, for example, to patients with mainly horizontal growth, presence of muscular balance and some freedom for incisors to tip forward.

In this study, no heat-activated nickel-titanium wires were used. Additionally, all study models tested were free of crowding. Moreover, the self-ligating brackets used herein were passive. In active self-ligating brackets, the more the diameter of wires increase, the greater the friction which can be higher than conventional brackets.^15^

Therefore, according to the present results and the growing development of self-ligating brackets systems, it is reasonable to assert that much has to evaluate with regards to stress produced by the use of self-ligating brackets compared to conventional brackets in expansive mechanics.

## CONCLUSION

Based on the results of the current study, it is suggested that both bracket systems produced stress when activated. However, conventional brackets produced greater stress in comparison to passive self-ligating brackets. Therefore, the present pilot study reveals that self-ligating brackets produce softer forces in periodontal tissues in alignment expansive mechanics.

## References

[1] Brito VS, Ursi WJS (2006). O aparelho pré-ajustado: sua evolução e suas
prescrições. Rev Dental Press Ortod Ortop Facial.

[2] Picchioni MS (2007). Análise comparativa dos níveis de atrito em braquetes e
autoligados [dissertação].

[3] Frank C (1980). A comparative study of frictional resistances between
orthodontic bracket and arch wire. Am J Orthod.

[4] Voudouris JC (1997). Interactive edgewise mechanisms: form and function
comparison with conventional edgewise brackets. Am J Orthod Dentofacial Orthop.

[5] Damon DH (1998). The Damon low-friction bracket: a biologically
compatible straight-wire system. J Clin Orthod.

[6] Maltagliati L (2009). Sistema autoligado: quebrando paradigmas. Ortodontia SPO.

[7] Rocha JET, Fuziy A, Tukasan PC, Oliveira RCG (2006). Fotoelasticidade: aplicabilidade na mecânica
ortodôntica. Braz Oral Res..

[8] Vuolo JH (1998). Polarização da luz e displays TN.

[9] Rossato C (1982). Estudo fotoelástico das áreas de pressão, produzidas no
periodonto, por forças ortodônticas, na distalização do canino pelos métodos
convencionais e com "Power arm". [dissertação].

[10] Glickman I, Roeber FW, Brion M, Pameijer JH (1970). Photoelastic analysis of internal stresses in the
periodontium created by occlusal forces. J Periodontol.

[11] Interlandi S (2002). Diagrama de contorneamento ortodôntico para a técnica do
arco contínuo (Straight Wire). Ortodontia.

[12] Dobranszki A (2009). Estudo fotoelástico do controle vertical com arco de
dupla chave na técnica Straight wire. Rev Dental Press Ortod Ortop Facial.

[13] Pandis N (2007). Self ligating vs conventional brackets in the treatment
of mandibular crowding: a prospective clinical trial of treatment duration and
dental effects. Am J Orthod Dentofacial Orthop.

[14] Kochenborger R (2009). Avaliação das alterações dentárias e do perfil facial obtidas
no tratamento ortodôntico com braquetes autoligáveis [dissertação].

[15] Lorenz M (2011). Active and passive self-ligation: a
myth?. Angle Orthod.

